# Antibiotic loaded β-tricalcium phosphate/calcium sulfate for antimicrobial potency, prevention and killing efficacy of *Pseudomonas aeruginosa* and *Staphylococcus aureus* biofilms

**DOI:** 10.1038/s41598-020-80764-6

**Published:** 2021-01-14

**Authors:** Nan Jiang, Devendra H. Dusane, Jacob R. Brooks, Craig P. Delury, Sean S. Aiken, Phillip A. Laycock, Paul Stoodley

**Affiliations:** 1 Department of Microbial Infection and Immunity, The Ohio State University Wexner Medical Center, Columbus, OH 43210 USA; 2 Department of Orthopaedics, Southern Medical University Nanfang Hospital, Guangzhou, 510515 Guangdong China; 3 Center for Clinical and Translational Research, The Ohio State University Nationwide Children’s Hospital, Columbus, OH 43205 USA; 4 Biocomposites Ltd., Keele Science Park, Keele, Staffordshire, ST5 5NL UK; 5 Department of Orthopaedics, The Ohio State University Wexner Medical Center, Columbus, OH 43210 USA; 6 National Centre for Advanced Tribology at Southampton (nCATS) and National Biofilm Innovation Centre (NBIC), Department of Mechanical Engineering, University of Southampton, Southampton, SO17 1BJ UK

**Keywords:** Immunology, Microbiology

## Abstract

This study investigated the efficacy of a biphasic synthetic β-tricalcium phosphate/calcium sulfate (β-TCP/CS) bone graft substitute for compatibility with vancomycin (V) in combination with tobramycin (T) or gentamicin (G) evidenced by the duration of potency and the prevention and killing efficacies of *P. aeruginosa* (PAO1) and *S. aureus* (SAP231) biofilms in in vitro assays. Antibiotic loaded β-TCP/CS beads were compared with antibiotic loaded beads formed from a well characterized synthetic calcium sulfate (CS) bone void filler. β-TCP/CS antibiotic loaded showed antimicrobial potency against PAO1 in a repeated Kirby-Bauer like zone of inhibition assay for 6 days compared to 8 days for CS. However, both bead types showed potency against SAP231 for 40 days. Both formulations loaded with V + T completely prevented biofilm formation (CFU below detection limits) for the 3 days of the experiment with daily fresh inoculum challenges (*P* < 0.001). In addition, both antibiotic loaded materials and antibiotic combinations significantly reduced the bioburden of pre-grown biofilms by between 3 and 5 logs (*P* < 0.001) with V + G performing slightly better against PAO1 than V + T. Our data, combined with previous data on osteogenesis suggest that antibiotic loaded β-TCP/CS may have potential to stimulate osteogenesis through acting as a scaffold as well as simultaneously protecting against biofilm infection. Future in vivo experiments and clinical investigations are warranted to more comprehensively evaluate the use of β-TCP/CS in the management of orthopaedic infections.

## Introduction

Orthopaedic implant associated infections, including periprosthetic joint infections (PJI) and fracture-related infections (FRI), still pose great challenges to orthopaedic surgeons. According to a recent study^1^, the 1-year and 5-year risks of PJI were 0.69% and 1.09% for total hip arthroplasty (THA), and 0.74% and 1.38% for total knee arthroplasty (TKA), respectively. As for FRI, it’s estimated that the FRI incidence ranges anywhere from 0.4 to 16.1%, with an average of 5%^2^. Despite many efforts, current clinical efficacy of PJI and FRI, especially regarding the incidence of infection relapse^3,4^, remains unsatisfactory, which is primarily attributed to the presence of difficult-to-eliminate biofilms^5,6^. Therefore, how to effectively treat the biofilms is the key to lower the risk of reinfections and improve treatment efficacy^7,8^.

Clinically, one of the most frequently used management strategies in orthopaedic device associated infections is radical debridement and wound pulse lavage (PL), followed by local implantation of antibiotic-loaded poly methylmethacrylate (PMMA) bone cement (ALBC) or absorbable mineral based materials such as calcium sulfate (CS). Previous work from our lab^9^ has shown improved efficacy following combinations of antibiotic-loaded CS and PL compared with these interventions alone, in removing *P. aeruginosa* and *S. aureus* biofilms attached to the surfaces of stainless steel. Although the primary function of ALBC is structural integrity in fixation or as a spacer, it can also be used in the form of beads with the primary function of local antibiotic release^10^. Compared with PMMA, CS beads has many advantages, such as carrying a wider range antibiotics such as those which may be heat sensitive, being completely biodegradable and not requiring a second surgery for removal^11^. Therefore, antibiotic-loaded CS (ALCS) has been used in the management of implant associated infections^12,13^.

In addition to infection management, absorbable based mineral materials or ALBC can also provide early protection against biofilm formation after primary osteoarticular surgery. Previous studies have indicated that local use of ALBC is effective in preventing both PJI^14–17^ and FRI^18–21^ following joint arthroplasty and open fracture surgery, respectively. After analyzing the prophylactic effect of ALBC against infection in primary cemented TKA of 731,214 cases, Jameson et al.^16^ concluded that ALBC was associated with a lower risk of revisions for infections (hazard ratio 0.84, 95% CI 0.67–1.01). As for FRI, a recent systematic review^22^ summarized the effect of local antibiotic prophylaxis when treating open limb fractures. Outcomes also showed a decreased FRI risk of 12% when additional local antibiotics were provided prophylactically. Whilst most of these studies focused on PMMA, clinical evidence of absorbable based mineral materials in primary prophylaxis of bone and joint surgery remains limited.

Absorbable mineral-based beads are primarily used as bone void fillers to manage dead space and promote bone growth. In a similar manner in which orthopaedic surgeons have mixed antibiotics into bone cement to add antimicrobial functionality to PMMA, antibiotics can also be added to mineral bone void fillers^23^. In previous in vitro studies, we have demonstrated synthetic pharmaceutical grade CS is compatible with a wide range of antibiotics used alone or in dual combinations^23^ and that the release of antibiotics at potent levels can be sustained over multiple weeks and can significantly prevent or retard the establishment of biofilms and eradicate or significantly reduce the numbers of bacteria in pre-established biofilms of Gram-negative and Gram-positive pathogens common to PJI^24–26^. Here we assessed the compatibility and potency of a novel biphasic absorbable material composed of 50% beta-tricalcium phosphate and 50% CS (β-TCP/CS) for controlling biofilms. This biphasic β-TCP/CS material has been designed to stimulate osteogenic activity and accelerate bone formation^27,28^. Here we assessed the antimicrobial potency when β-TCP/CS in the form of beads were loaded with vancomycin (V) in combination with tobramycin (T) or gentamicin (G). We compared antibiotic-loaded β-TCP/CS beads with the well characterized antibiotic-loaded CS beads in potency duration and the prevention and killing efficacy of *P. aeruginosa* and *S. aureus* biofilms.

## Materials and methods

### Bacterial strains and culture conditions

*P. aeruginosa* wild type PAO1^29^ and *S. aureus* SAP231 (a bioluminescent transformed USA 300 strain)^30^ were used in this study. PAO1 and SAP231 were grown in Tryptic Soy Broth (TSB; Sigma-Aldrich, St. Louis, MO) and Brain Heart Infusion broth (BHI; Sigma-Aldrich, St. Louis, MO), respectively, at 37˚C overnight on shaker conditions set at 200 rpm. The minimum inhibitory concentration (MIC) of vancomycin (V), tobramycin (T) and gentamicin (G) was determined by microbroth dilution^31^ using a 96 well-plate and two-fold dilutions from 0.125 to 64 for T and G and up to 1024 for V. OD_600_ was measured by plate reader and the concentration at which the OD was < 0.1 used as the breakpoint MIC value. The MIC of V,T and G against SAP231 was 4.0, 32.0 and 32.0 µg/mL respectively. The MIC of T and G against PAO1 were 4.0 μg/mL, but this strain was highly resistant to vancomycin (V) with a MIC > 1024 μg/mL.

### Preparation of the antibiotic-loaded beads (ALB)

Antibiotic-loaded Beads (ALB) were prepared using Genex and Stimulan Rapid Cure products (Biocomposites Ltd., Staffordshire, UK). Genex is a synthetic biphasic material composed of β-TCP and CS in a weight ratio of 1:1, and Stimulan is a high purity CS product. The mixture ratio for V + T and V + G groups were 1000 mg vancomycin and 240 mg tobramycin, and 1000 mg vancomycin and 240 mg gentamicin, per 10 cc (~ 16 g) Genex or Stimulan (~ 20 g) powder, respectively, for clinical relevance^32^. All antibiotics were obtained from GoldBio (St. Louis, MO). The procedures for preparation of the beads have been described previously^9^. The beads were 4.8 mm in diameter approximately 0.108 g in weight, and each antibiotic-loaded bead would be expected to contain on average 4.13 mg of vancomycin and 1.02 mg of tobramycin or gentamicin.

### Antibiotic-elution potency and duration for inhibition of planktonic bacteria

A revised Kirby-Bauer test, as described previously^24^, was used to determine the potency of ALB over time. Overnight cultures of PAO1 and SAP231 were diluted to 1% in TSB and BHI broth, respectively. 100 µL of diluted culture was then spread on either Tryptic Soy Agar (TSA) or BHI agar plates, followed by centrally placing ALB with either V + T or V + G on top of the agar with sterile forceps. The plates were incubated at 37˚C and 5% CO_2_ for 24 h and zones of inhibition (ZOI) were subsequently analyzed and recorded. Subsequently, the beads were then transferred to a newly prepared bacterial lawn. The procedure was repeated each day until the ZOI was zero. The maximum diameter of the ZOI was measured every 24 h with a ruler, and then converted into units of area (cm^2^) using the formula A = πr^2^. Where A is the area of the circle, r is the radius and π is a constant (approximately equals to 3.14159). Area was used rather than diameter since heterogeneity in the beads can sometimes lead to irregular shaped ZOI.

### Prevention of biofilm formation

ALB and unloaded beads (10 beads per well) were placed into 6-well plates (Falcon, Corning, NY) and 35 mm MatTek tissue culture plates (MatTek Corporation, Ashland, MA). We used the 35 mm MatTek plates specifically for those biofilms in the prevention assay to be imaged on the confocal microscope to be compatible with our stage holder. Each well was inoculated with 4 mL of an overnight culture diluted with fresh TSB for PAO1 or BHI for SAP231 to achieve a concentration of approximately 10^6^ CFU/mL and incubated on a shaker set at 37 °C and 50 rpm. Every 24 h, the spent media were replaced, and the beads were subjected to a fresh bacterial challenge of 2 mL of 10^6^ CFU/mL.

### Killing efficacy of established biofilms

Initially, each well of a 6-well plate was inoculated with either 4 mL of TSB and ~ 10^6^ CFU/mL of PAO1 or 4 mL BHI and ~ 10^6^ CFU/mL SAP231. Biofilms were allowed to establish for 3 days (37 °C and 50 rpm) with daily media exchanges of 2 mL fresh media. After 72 h, 10 ALB or unloaded beads per well were added to each plate along with fresh media. Every 24 h, the spent media was replaced. The effect of the beads on the amount of biofilm was assessed by viable count using the plate count method.

### Viable cell count (CFU)

Individual wells were rinsed twice with 4 mL phosphate buffered saline (PBS) (Dulbecco’s, Gibco, Grand Island, NY) to remove planktonic cells. The surface of each well was scraped using a cell scraper (Corning, NY) to remove the attached biofilms into 1 mL PBS. Samples were vortexed for 20 s to homogenize the biofilm bacteria, tenfold serial dilutions were performed in PBS and plated onto TSA (for PAO1) and BHI (for SAP231) agar plates using the drop plate method, in triplicate^33^. Wells with bacteria-inoculated media without any beads (control group 1, C1) or unloaded beads (control group 2, C2) were used as negative controls.

### Confocal laser scanning microscopy (CLSM)

To support the CFU data from the prevention assay, CLSM (Olympus FV10i, Waltham, MA) was performed on biofilms growing in the MatTek plates. At days 1 and 3, using the fluorescent stain SYTO9 (Thermo Fisher Scientific, USA), which stains both live and dead cells green to microscopically quantify and statistically compare the biofilm surface area. Each plate was rinsed with PBS (as detailed in the viable cell count) and stained with 1 µl of SYTO9 per 1 mL of PBS for 20 min, according to manufacturer’s instructions. Then, the plates were gently rinsed with PBS once more and finally 1 mL of PBS was added to the well before observation. In each group, three CLSM images were selected and ImageJ (NIH) software was used to measure the surface areas of the biofilms.

### Statistical analysis

Statistical analysis was conducted using the Statistical Product and Service Solutions (SPSS) software (version 19.0) (SPSS Inc., Chicago, IL). The CFU data was Log_10_ transformed and the student* t* test or one-way analysis of variance (ANOVA) was firstly used to evaluate the effect of single independent factor on the CFU count. F-test was used in case of homoscedasticity, and Bonferroni method was used for subsequent post-hoc multiple comparisons. While Welch test and Dunnett’s T3 method was applied, respectively in case of heteroscedasticity. Then, multivariate analysis of variance (MANOVA) was used to investigate the influences of treatment, exposure time, bacteria strain and antibiotic carrier on CFU count as well as potential interactions among the four independent factors, sorted by the prevention and killing groups, respectively. Differences were considered statistically significance when* P* ≤ 0.05.

## Results

### Antibiotic-elution potency and duration for inhibition of planktonic bacteria

**Figure 1:**
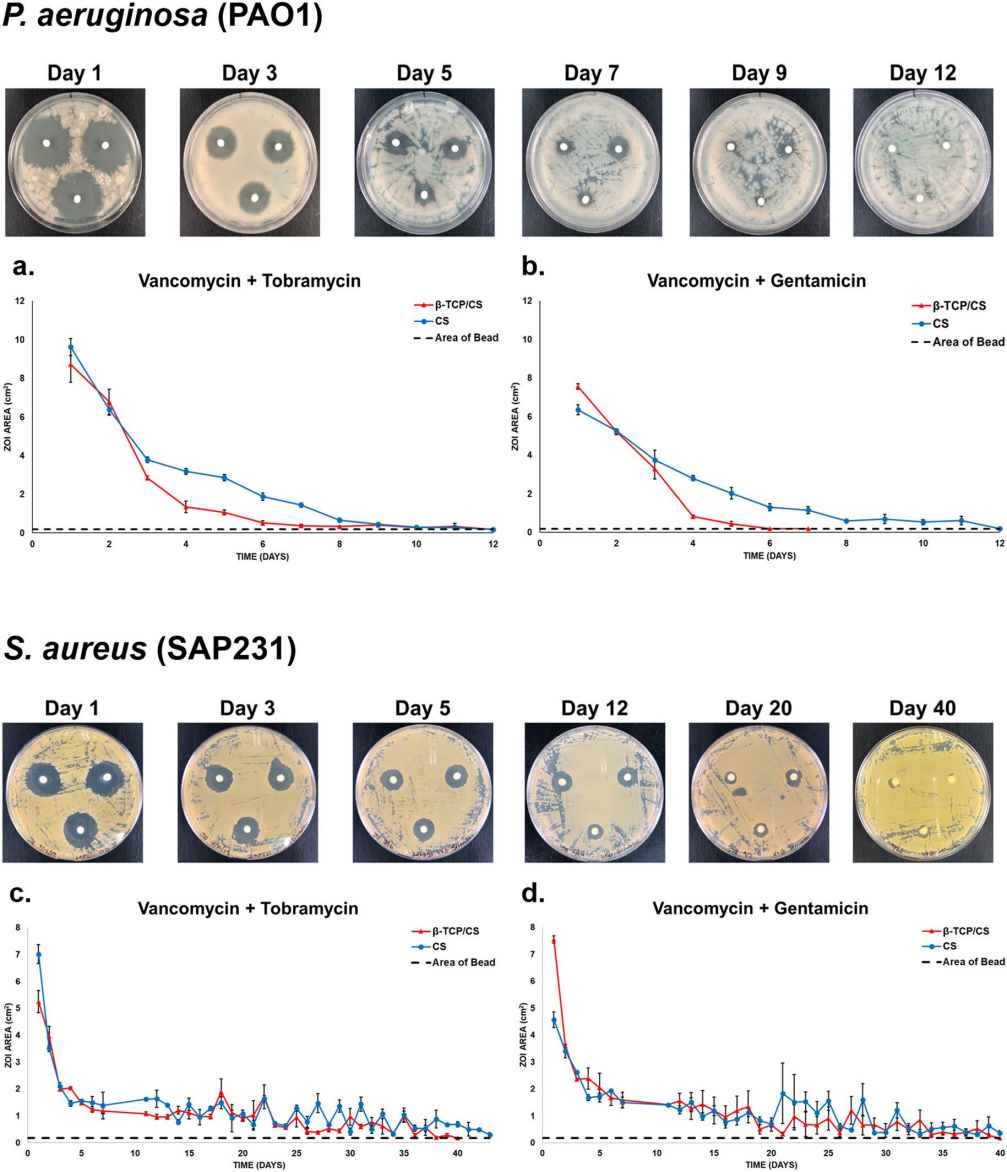
Repeat zones of inhibition (ZOI) of biofilm assays of PAO1 and SAP231, showing the sizes of the ZOI (in cm^2^) from antibiotic loaded β-TCP/CS and CS beads over time. Images are representative photographs showing the ZOI from the two bacterial strains observed on agar plates at various days throughout the study. Assays were performed in triplicate, and data are expressed as the means of 3 data points ± standard error (SE).

PAO1 was susceptible to both V + T and V + G released from CS and β-TCP/CS beads, producing a large ZOI after 1 day (Fig. 1a, b). β-TCP/CS showed potency for 8 and 5 days against PAO1 for V + T and V + G respectively, while CS demonstrated extended potency for up to 12 days. Against SAP231, CS and β-TCP/CS showed similar potency profiles with both antibiotic combinations, with an initial burst over the first 2–3 days, followed by sustained potency up to approximately 40 days (Fig. 1c, d).

### Prevention efficacy of biofilm formation

**Figure 2:**
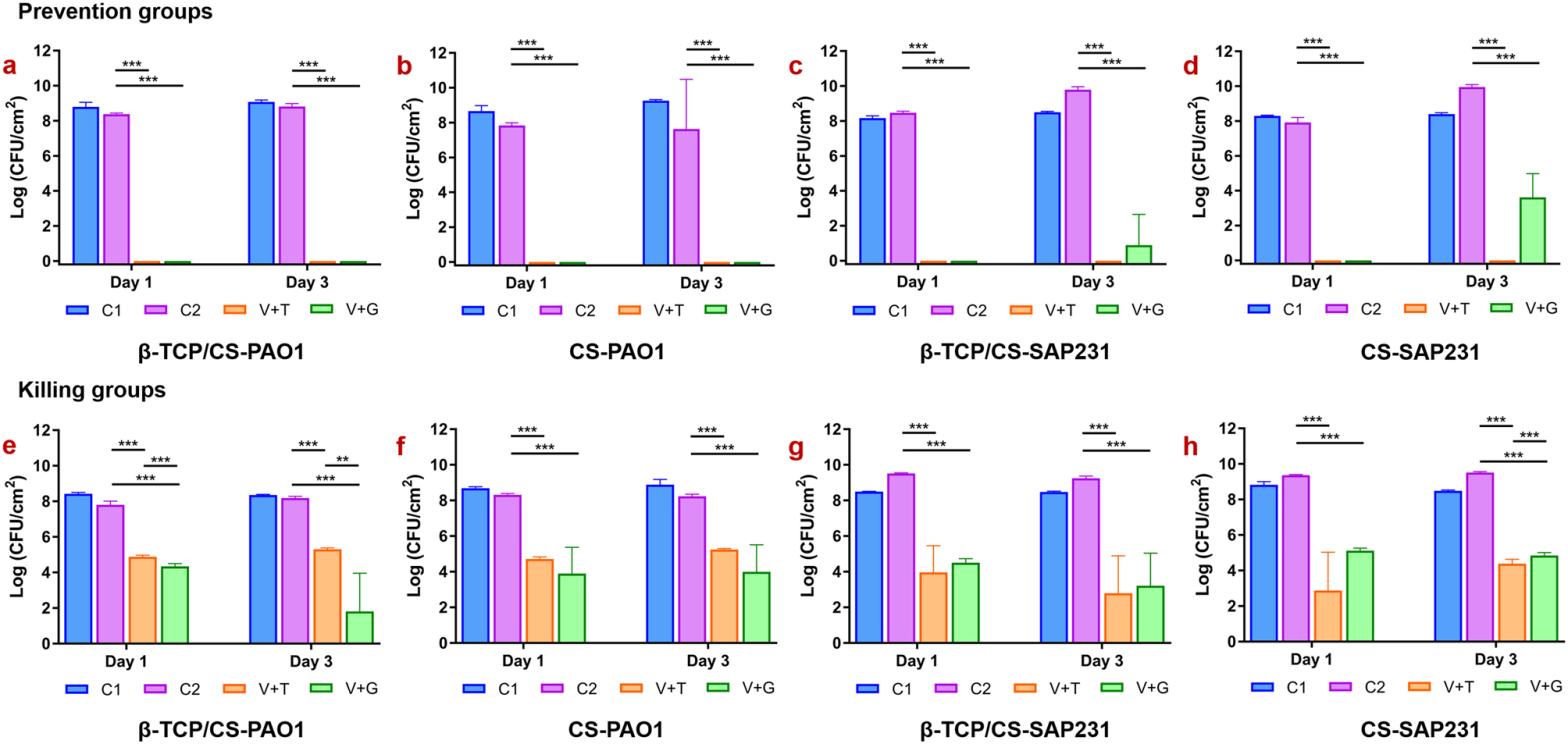
Comparisons of the *P. aeruginosa* PAO1 and *S. aureus* SAP231 CFU counts between different types in the prevention and killing groups at day 1 and day 3. Data are expressed as means of 9 data points with standard deviation bars. (Prevention groups: (**a**)–(**d**); Killing groups: (**e**)–(**g**). C1 represents blank control without beads, C2 represents controlled group with unloaded beads; *P < 0.05, **P < 0.01, ***P < 0.001).

**Figure 3:**
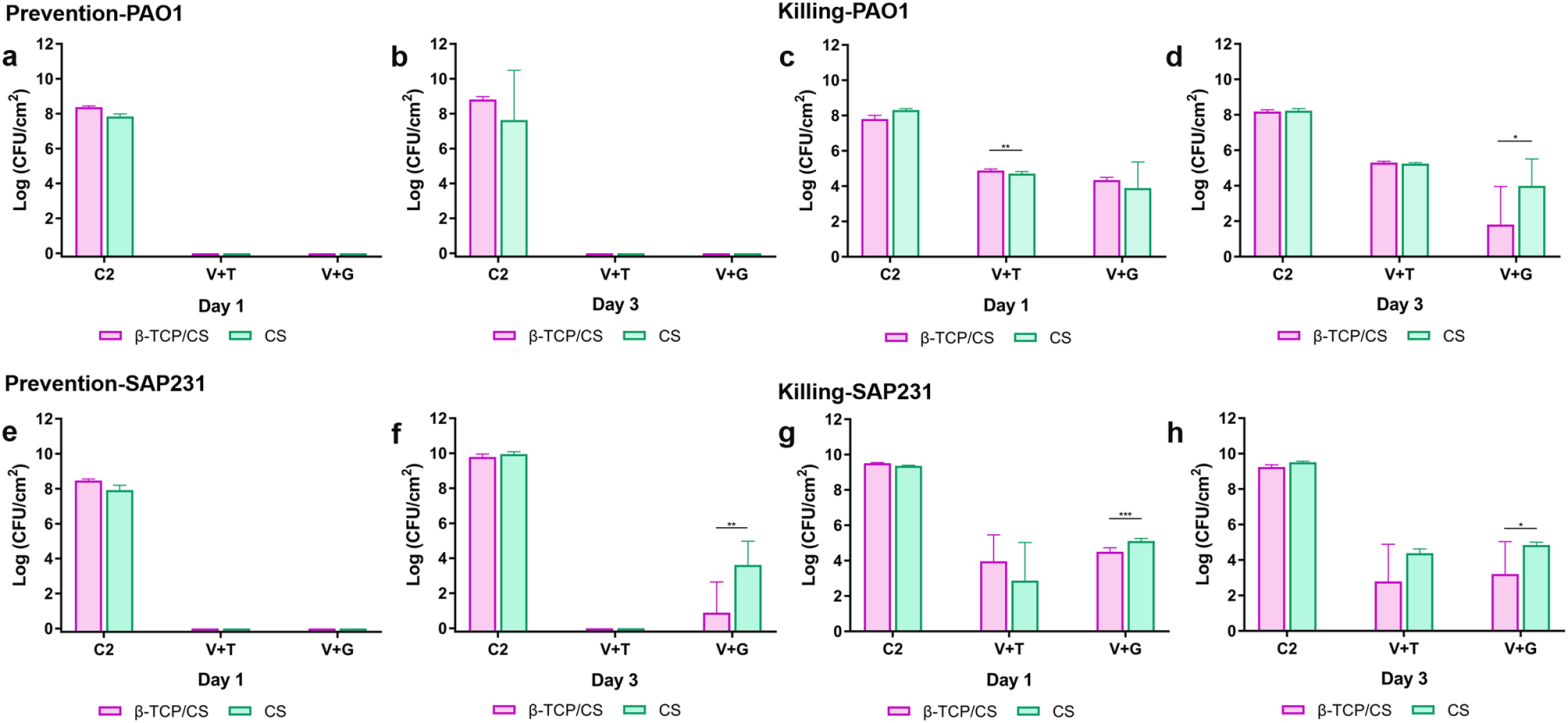
Comparisons of the *P. aeruginosa* PAO1 and *S. aureus* SAP231 CFU counts following treatment with β-TCP/CS or CS beads. Data expressed as means of 9 data points with standard deviation bars. (PAO1: (**a**)–(**d**); SAP231: (**e**)–(**h**). C2 represents controlled group with unloaded beads; *P < 0.05, **P < 0.01, ***P < 0.001).

In the biofilm prevention assays, both β-TCP/CS and CS beads loaded with the antibiotic combinations reduced PAO1 biofim formation below CFU detection limits (*P* < 0.001) with between approximately 8 and 9-log reductions for days 1 and 3 with both antibiotic combinations compared to the controls (Figs. 2a, b; 3a, b; Supplementary Table S1). Similar results were seen againt SAP231 with V + T, although colonies were recovered from both materials loaded with the V + G combination after 3 days, with β-TCP/CS resulting in an approximate 8 log reduction, which was significantly greater than the 6 log reduction achieved by CS ALB (*P* < 0.01) (Figs. 2c, d; 3e, f, Supplementary Table S1). The overall comparison outcome of the MANOVA model was significant (*P* < 0.001). However, the antibiotic carrier showed no significant effect (*P* = 0.558), but the other three factors (treatment, exposure time and bacteria strain) revealed significant contributions to the CFU count. In addition to their independent effect on the CFU count, all of the four factors (treatment, exposure time, bacteria strain and antibiotic carrier) demonstrated a significant interaction effect on the CFU count (Supplementary Table S2).

### Killing efficacy of the established biofilms

In the biofilm killing assays, β-TCP/CS and CS beads loaded with the various antibiotic combinations significantly reduced the amount of viable bacteria in the PAO1 biofilms by approximately 3 to 4 logs after 24 h exposure to V + T (*P* < 0.001) with similar reductions observed after 72 h (Figs. 2e, f; 3c, d; Supplementary Table S1). ALBs combined with V + G showed an increased reduction compared to V + T and, β-TCP/CS beads at both 24 and 72 hs exposure showed statistically significant reductions between the two antibotic combinations (*P* < 0.01) (Fig. 2e). Killing efficacy against SAP231 biofilms showed similar log reductions to those against PAO1 and these were statistically significant compared to controls (*P* < 0.01) (Fig. 2g, h). SAP231 killing was indifferent and non-significant with V + T at days 1 and 3 with both the carrier biomaterials, however antibiotics V + G loaded in β-TCP had significant killing effect on biofilms as compared to CS (Fig. 3g, h). Moreover, based on the ANOVA, there was no obvious trend with regards to exposure time or antibiotic combinations. The overall comparison outcome of the MANOVA model was also significant (*P* < 0.001), but only the treatment strategy and antibiotic carrier contributed significantly to the CFU count (*P* < 0.001) individually. In addition, significant interactions were found between treatment with exposure time, treatment with bacteria strain, treatment with antibiotic carrier, and exposure time with antibiotic carrier. Moreover, such significant interactions were also observed among treatment, exposure time and antibiotic carrier, as well as among all of the four independent factors (Supplementary Table S2).

### Confocal laser scanning microscopy (CLSM)

**Figure 4:**
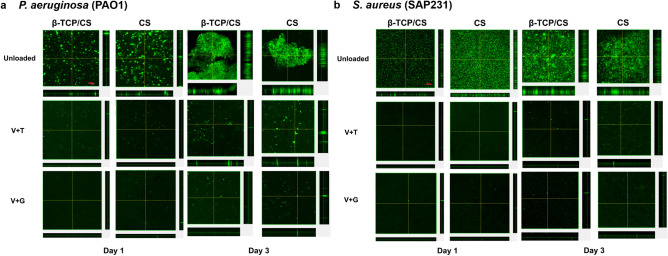
Prevention of *P. aeruginosa* PAO1 (**a**) and *S. aureus* SAP231 (**b**) biofilm formations on the glass surface of a MatTek tissue culture plate at days 1 and 3. Red scale bar (top left panel): 50 μm.

**Figure 5:**
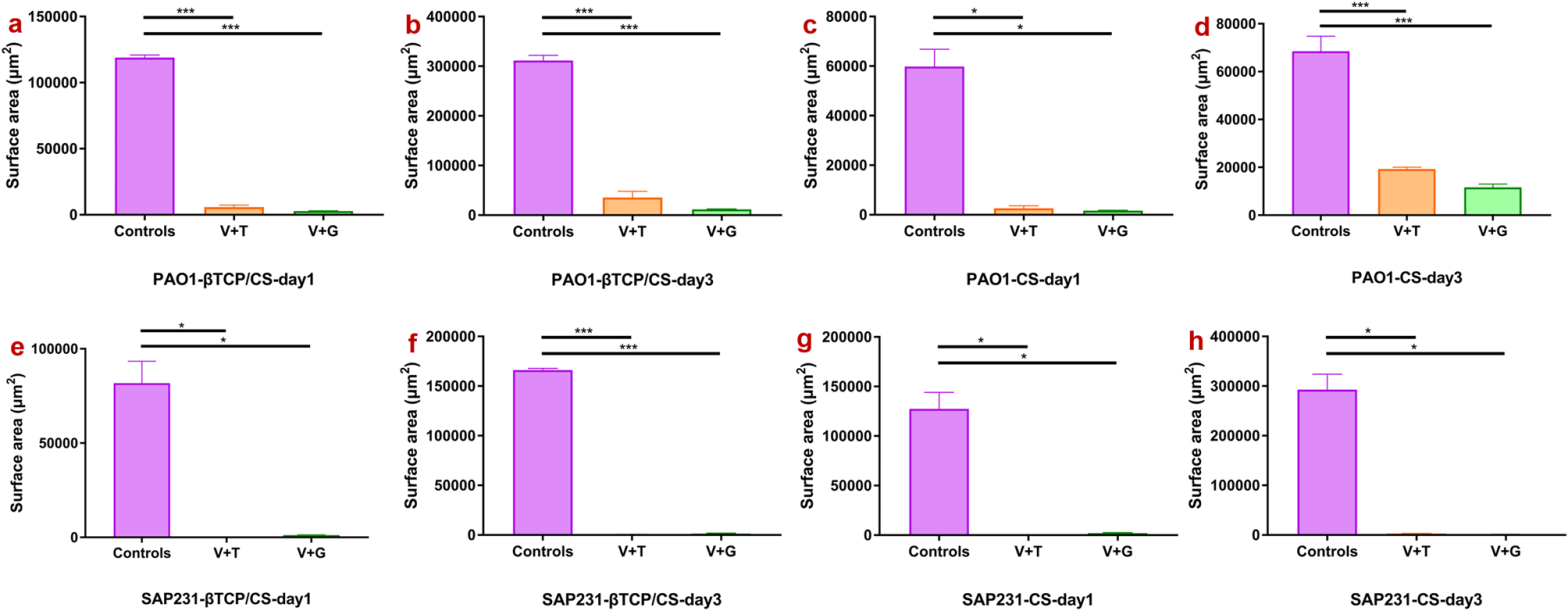
Surface area of *P. aeruginosa* PAO1 and *S. aureus* SAP231 biofilms in the prevention groups. The mean surface areas of PAO1 and SAP231 biofilms following V + T and V + G prophylaxic interventions at day 1 and day 3 were significantly lower than those of the respective controls. *P < 0.05, **P < 0.01, ***P < 0.001.

In general, the confocal images corroborated the CFU counts in the biofilm prevention assays. Both β-TCP/CS and CS beads, either mixed with V + T or V + G, successfully reduced PAO1 (Fig. 4a) and SAP231 (Fig. 4b) biofilm formation for at least 3 days. Outcomes of surface area analyses also confirmed significantly descreased formations of PAO1 and SAP231 biofilms by prophylaxic interventions of both V + T and V + G loaded β-TCP/CS and CS beads Fig. 5).

## Discussion

In vitro assays are useful to assess antibiotic compatibility, potency and release kinetics from PMMA as well as other mineral based bone void fillers used in orthopaedic surgery for infection control in PJI and FRI, in the context of managing bacterial biofilms, a complicating factor in these infections. Previously, we have used in vitro methods to demonstrate potency and duration of release of a wide variety of antibiotics from CS beads against planktonic and biofilm cultures^23–26^. Here, we used these methods to assess such antimicrobial efficacy of antibiotics released from a fully synthetic biphasic bone graft substitute composed of β-TCP and CS (β-TCP/CS) to assess how this formulation compared to that of synthetic CS. We combined two frequently used antibiotic combinations used in bone cements or mineral based bone void fillers, vancomycin, tobramycin and gentamicin^34^, and we also selected common representatives of Gram positive and negative isolated bacteria from clinical infection cases, *S. aureus* and *P. aeruginosa*^35^ as challenge pathogens to assess efficacy of antibiotic loaded β-tricalcium phosphate/calcium sulfate (β-TCP/CS) beads. The performance of V + T by CS beads against PAO1 was similar to our previous study^25^ in terms of the duration and potency of the beads which showed efficacy for 8 days. Similarly, the ZOI profile with SAP231 was in accordance with previously published data^25^. Comparing the results of β-TCP/CS to those of CS beads in the current study suggests that β-TCP/CS beads possesses similar levels and durations of antimicrobial activity against two pathogens common to PJI and FRI in both planktonic and biofilm phenotypes. However, there were some differences, β-TCP/CS beads loaded with antibiotics displayed reduced potency against *P. aeruginosa* PAO1 after 4 days compared to CS and potency only extended to 6 days. This relative difference in tobramycin potency may be related to the amount of CS in the material (100% vs 50%) which is more absorbable and has a lower porosity than beta-tricalcium phosphate^36^ Another possibility is that *P. aeruginosa* may produce metabolites which interact with the materials in different ways thus influencing potency. This phenomenon is likely not general to all Gram negative bacteria since we saw potency of V + T released from CS beads against strains of *K. pneumoniae* and *A. baumannii* for 40 days^25^, similar to that of staphylococcal strains. It is therefore not unreasonable to speculate that antibiotic loaded β-TCP/CS will show greater potency duration against Gram negative strains other than *P. aeruginosa*. Nevertheless, in our biofilm challenges we saw significant reductions in *P. aeruginosa* over 3 days of exposure to both bead types. β-TCP/CS or CS beads mixed with V + T and V + G resulted in completed killing of *P. aeruginosa* PAO1 at days 1 and 3, demonstrating an in vitro prophylactic effect against biofilm formation. This outcome was similar to our previous study^25^, which also revealed that combinations of V + T loaded by CS could prevent the PAO1 biofilm formation for at least 3 days. As for *S. aureus*, both β-TCP/CS and CS beads containing V + T were also able to prevent SAP231 biofilm formation at days 1 and 3, while V + G showed complete prevention at day1 but allowed some growth after 3 days. It is important to note that this assay represents 3 heavy inoculations as well as removal of any antibiotics that might have built up over the proceeding 24 h and thus can be considered a robust challenge.

Although both formulations demonstrated similar potency and antimicrobial activity against biofilms some differences might be explained to differences in mineral chemical and physical compositions. Bone graft substitute, β-TCP and CS (β-TCP/CS) has a finer microarchitecture than CS such that the porosity more closely resembles cancellous bone, thus allowing more rapid absorption than hydroxyapatite (HA)^37^. β-TCP also shows a good biocompatibility^38^ and faster osseointegration with restoration of physiological architecture compared to HA^39,40^. The β-TCP/CS has both bioactive and biphasic properties, with CS acting as a barrier to prevent initial in-growth of the soft tissue and β-TCP acting as a longer term scaffold. The negatively charged surface can stimulate the osteogenic activity, accelerate bone formation and fusion by harnessing key proteins, and direct osteoprogenitor cell proliferation and osteoblast adhesion for rapid osteogenesis^27,28,41^. Our data suggests that β-TCP/CS may also have functionality for releasing antibiotics locally in high enough concentrations and for long enough periods to significantly prevent or reduce existing biofilms as well as providing a longer term osteoconductive scaffold. However, it is also important to consider long term exposure of antibiotics on the development of resistance, particularly since *P. aeruginosa* is known to develop resistance against aminoglycosides^42^. There is debate regarding the use of high concentrations of antibiotics in the joint space, on one hand it is argued that if concentrations are high enough for long enough where biofilms are present all bacteria will be killed then there is no chance of developing resistance and there is a greater chance when relying on systemic administration alone where local concentrations are likely not going to reach levels where biofilms can be eradicated^43^. On the other hand there is not enough clinical data to assess the benefit of locally high concentrations in light of the fear of antimicrobial resistance (AMR)^44^. In ongoing experiments we are assessing the probability of the development of AMR of antibiotics alone and in combination released from β-TCP/CS and CS beads against our test strains. Also in future work it is important to assess the impact that antibiotics might have on the absorption kinetics with respect to the duration of stability of the scaffold.

## Conclusions

In summary, the present study demonstrated that vancomycin, tobramycin and gentamicin retain potency when mixed and set in β-TCP/CS and release these antibiotics over periods of up to 40 days. Moreover, β-TCP/CS displayed similar efficacy with CS beads in both prevention and significantly reducing bioburden of PAO1 and SAP231 biofilms, suggesting that this material when loaded with antibiotics has potential to stimulate osteogenesis through acting as a scaffold and simultaneously protecting against biofilm infection. However, future in vivo experiments and clinical investigations are warranted to more comprehensively evaluate this use of β-TCP/CS in the management of orthopaedic infections.

## Supplementary Information

Supplementary Tables.

## Abbreviations

β-TCP/CS: β-Tricalcium phosphate/calcium sulfate
V: Vancomycin
T: Tobramycin
G: Gentamicin
CS: Calcium sulfate
PJI: Periprosthetic joint infection
FRI: Fracture-related infection
THA: Total hip arthroplasty
TKA: Total knee arthroplasty
PL: Pulse lavage
PMMA: Poly-methyl methacrylate
ALBC: Antibiotic loaded bone cement
ALCS: Antibiotic-loaded calcium sulfate
TSB: Trypticase Soy Broth
BHI: Brain Heart Infusion
MIC: Minimum inhibitory concentration
ALB: Antibiotic-loaded beads
TSA: Either Tryptic Soy Agar
ZOI: Zones of inhibition
PBS: Phosphate buffered saline
CLSM: Confocal laser scanning microscopy
SPSS: Statistical Product and Service Solutions
ANOVA: Analysis of variance
MANOVA: Multivariate analysis of variance
