# Supplementary material for: Antibiotic loaded β-tricalcium phosphate/calcium sulfate for antimicrobial potency, prevention and killing efficacy of Pseudomonas aeruginosa and Staphylococcus aureus biofilms
Source: Sci Rep. 2021 Jan 14;11:1446. doi: 10.1038/s41598-020-80764-6 (PMC7809051; doi:10.1038/s41598-020-80764-6)
Supplement: Supplementary file 1 — Supplementary Tables. [file 41598_2020_80764_MOESM1_ESM.docx]

**Antibiotic Loaded β-Tricalcium Phosphate/Calcium Sulfate For Antimicrobial Potency, Prevention And Killing Efficacy Of *Pseudomonas aeruginosa* And *Staphylococcus aureus* Biofilms**

Nan Jiang ^1, 2*^, Devendra H. Dusane ^3*^, Jacob R. Brooks ^1^, Craig P. Delury ^4^, Sean S. Aiken ^4^, Phillip A. Laycock ^4^, Paul Stoodley ^1, 5, 6, †^

1. Department of Microbial Infection and Immunity, The Ohio State University Wexner Medical Center, Columbus, OH 43210, USA.
2. Department of Orthopaedics, Southern Medical University Nanfang Hospital, Guangzhou, Guangdong 510515, China.
3. Center for Clinical and Translational Research, The Ohio State University Nationwide Children’s Hospital, Columbus, OH 43205, USA.
4. Biocomposites Ltd., Keele Science Park, Keele, Staffordshire ST5 5NL, UK.
5. Department of Orthopaedics, The Ohio State University Wexner Medical Center, Columbus, OH 43210, USA.
6. National Centre for Advanced Tribology at Southampton (nCATS) and National Biofilm Innovation Centre (NBIC), Department of Mechanical Engineering, University of Southampton, Southampton SO17 1BJ, UK.

*Nan Jiang and Devendra H. Dusane contributed equally to this study.

^†^ Correspondence to: Paul Stoodley

E-mail: Paul.Stoodley@osumc.edu

Tel: +1-614-292-7826

ORCID: 0000-0001-6069-273X

**SUPPLEMENTARY MATERIAL**

Table S1. The CFU counts of PAO1 and SAP231 biofilms following different interventions

| **Products** | **Duration** | **Control 1**  **(CFU/cm^2^)** | **Control 2**  **(CFU/cm^2^)** | **V+T**  **(CFU/cm^2^)** | **V+G**  **(CFU/cm^2^)** |
| --- | --- | --- | --- | --- | --- |
| **PAO1-Prevention groups** | | | | | |
| **β-TCP/CS** | Day 1 | 8.79 ± 0.27 | 8.38 ± 0.07 | 0.00 ± 0.00 | 0.00 ± 0.00 |
|  | Day 3 | 9.08 ± 0.11 | 8.81 ± 018 | 0.00 ± 0.00 | 0.00 ± 0.00 |
| **CS** | Day 1 | 8.66 ± 0.32 | 7.84 ± 0.15 | 0.00 ± 0.00 | 0.00 ± 0.00 |
|  | Day 3 | 9.26 ± 0.06 | 7.63 ± 2.86 | 0.00 ± 0.00 | 0.00 ± 0.00 |
| **PAO1-Killing groups** | | | | | |
| **β-TCP/CS** | Day 1 | 8.42 ± 0.08 | 7.80 ± 0.22 | 4.88 ± 0.10 | 4.35 ± 0.16 |
|  | Day 3 | 8.36 ± 0.04 | 8.18 ± 0.11 | 5.30 ± 0.08 | 1.81 ± 2.15 |
| **CS** | Day 1 | 8.69 ± 0.09 | 8.31 ± 0.08 | 4.72 ± 0.11 | 3.89 ± 1.48 |
|  | Day 3 | 8.88 ± 0.30 | 8.23 ± 0.12 | 5.24 ± 0.06 | 3.99 ± 1.52 |
| **SAP231-Prevention groups** | | | | | |
| **β-TCP/CS** | Day 1 | 8.16 ± 0.13 | 8.47 ± 0.09 | 0.00 ± 0.00 | 0.00 ± 0.00 |
|  | Day 3 | 8.50 ± 0.05 | 9.79 ± 0.17 | 0.00 ± 0.00 | 0.89 ± 1.76 |
| **CS** | Day 1 | 8.29 ± 0.04 | 7.92 ± 0.28 | 0.00 ± 0.00 | 0.00 ± 0.00 |
|  | Day 3 | 8.40 ± 0.08 | 9.95 ± 0.14 | 0.00 ± 0.00 | 3.62 ± 1.36 |
| **SAP231-Killing groups** | | | | | |
| **β-TCP/CS** | Day 1 | 8.49 ± 0.03 | 9.52 ± 0.04 | 3.96 ± 1.50 | 4.50 ± 0.23 |
|  | Day 3 | 8.47 ± 0.05 | 9.24 ± 0.13 | 2.79 ± 2.10 | 3.21 ± 1.83 |
| **CS** | Day 1 | 8.82 ± 0.18 | 9.37 ± 0.04 | 2.87 ± 2.16 | 5.11 ± 0.15 |
|  | Day 3 | 8.48 ± 0.05 | 9.51 ± 0.06 | 4.39 ± 0.25 | 4.85 ± 0.17 |

Control 1: without beads; Control 2: with unloaded beads;

β-TCP/CS: β-tricalcium phosphate/calcium sulfate; CS: calcium sulfate.

V+T: vancomycin and tobramycin; V+G: vancomycin and gentamicin

Data presented as mean ± standard deviation.

Table S2. Influences of treatment, exposure time, bacteria strain and antibiotic carrier on the CFU count and potential interactions among the four independent factors from multivariate analysis of variance (MANOVA).

| **Item** | **Prevention group** | | **Killing Group** | |
| --- | --- | --- | --- | --- |
|  | Test statistics (F) | *P* value | Test statistics (F) | *P* value |
| Corrected model | 393.288 | **＜0.001** | 70.882 | **＜0.001** |
| Treatment | 3938.856 | **＜0.001** | 659.935 | **＜0.001** |
| Exposure time | 58.788 | **＜0.001** | 2.833 | 0.094 |
| Bacteria strain | 20.396 | **＜0.001** | 2.389 | 0.123 |
| Antibiotic carrier | 0.345 | 0.558 | 13.602 | **＜0.001** |
| Treatment * Exposure time | 11.199 | **＜0.001** | 7.757 | **＜0.001** |
| Treatment * Bacteria strain | 26.954 | **＜0.001** | 37.335 | **＜0.001** |
| Treatment * Antibiotic carrier | 10.415 | **＜0.001** | 4.136 | **0.007** |
| Exposure time * Bacteria strain | 34.309 | **＜0.001** | 0.156 | 0.693 |
| Exposure time * Antibiotic carrier | 5.512 | **0.020** | 14.866 | **＜0.001** |
| Bacteria strain * Antibiotic carrier | 10.763 | **0.001** | 0.052 | 0.821 |
| Treatment * Exposure time * Bacteria strain | 15.326 | **＜0.001** | 0.718 | 0.542 |
| Treatment * Exposure time * Antibiotic carrier | 4.770 | **0.003** | 5.532 | **0.001** |
| Treatment * Bacteria strain * Antibiotic carrier | 4.586 | **0.004** | 0.575 | 0.632 |
| Exposure time * Bacteria strain * Antibiotic carrier | 8.370 | **0.004** | 0.634 | 0.427 |
| Treatment * Exposure time * Bacteria strain * Antibiotic carrier | 5.710 | **0.001** | 4.944 | **0.002** |

Treatment: blank controls (C1), unloaded beads (C2), V+T, and G+V.

Exposure time: day 1 and day 3.

Bacteria strain: *P. aeruginosa* PAO1 and *S. aureus* SAP 231

Antibiotic carrier: β-TCP/CS beads, and CS beads
